# Electrochemical Study of a Hybrid Polymethyl Methacrylate Coating using SiO_2_ Nanoparticles toward the Mitigation of the Corrosion in Marine Environments

**DOI:** 10.3390/ma12193216

**Published:** 2019-10-01

**Authors:** José Maya-Cornejo, Francisco J. Rodríguez-Gómez, Gustavo A. Molina, Juan Galindo-de-la-Rosa, Janet Ledesma-García, Ángel R. Hernández-Martínez, Rodrigo Esparza, Ramiro Pérez, Miriam Estévez

**Affiliations:** 1 Centro de Física Aplicada y Tecnología Avanzada, Universidad Nacional Autónoma de Mexico, Boulevard Juriquilla 3001, Santiago de Querétaro 76230, Qro., Mexico; iqm_jamc@yahoo.com.mx (J.M.-C.); gamol@fata.unam.mx (G.A.M.); arhm@fata.unam.mx (Á.R.H.-M.); resparza@fata.unam.mx (R.E.); 2 Departamento de Ingeniería Metalúrgica, Facultad de Química, Universidad Nacional Autónoma de Mexico, Ciudad Universitaria 04510, Mexico D.F., Mexico; fxavier@servidor.unam.mx; 3 Centro de Investigación y Desarrollo Tecnológico en Electroquímica, Pedro Escobedo 76703, Qro., Mexico; juandedios_galindo@hotmail.com; 4 Facultad de Ingeniería, Universidad Autónoma de Querétaro, Centro Universitario Cerro de las Campanas, Santiago de Querétaro 76010, Qro., Mexico; janet.ledesma@uaq.mx; 5 Instituto de Ciencias Físicas, Universidad Nacional Autónoma de Mexico, Av. Universidad s/n, Cuernavaca 62210, Mor., Mexico; ramirop21@gmail.com

**Keywords:** PMMA hybrid coating, SiO_2_ nanoparticles, scanning electrochemical microscopy, electrochemical impedance spectroscopy

## Abstract

The demand for hydrophobic polymer-based protective coatings to impart high corrosion resistance has increased recently. The increase of the hydrophobicity in a hybrid coating is a new challenge, for that reason and in order to protect a metallic surface of oxidant agents, a poly (methyl methacrylate) (PMMA) coating with the addition of a different amount of silicon dioxide (SiO_2_) was developed. The hybrid coating was applied on a sample of stainless steel AISI 304 by the dip-coating method. The characterization of the coatings was determined by electrochemical impedance spectroscopy and with a scanning electrochemical microscopy. The best coatings were PMMA and PMMA + SiO_2_ 0.01% that exhibits a real impedance in the Nyquist diagram of 760 and 427,800 MΩ⋅cm^2^, respectively, and the modulus of the real impedance in the Bode diagram present values of 2.2 × 10^8^ and 3.3 × 10^8^ Ω⋅cm^2^. Moreover, the phase angle presents constant values around 75° to 85° and 85° for the PMMA and PMMA + SiO_2_ 0.01%, respectively. Moreover, the values of the real resistance for the PMMA + SiO_2_ 0.01% coating present values in the order of Mega-ohms despite the coating exhibits an artificial defect in their surface. The contact angle test showed that the hydrophobicity of the hybrid PMMA + SiO_2_ 0.01% coating is higher than that of the pure PMMA coatings. The hybrid PMMA + SiO_2_ coatings developed in this work are a very interesting and promising area of study in order to develop efficient products to protect metallic surfaces from corrosion phenomenon.

## 1. Introduction

Materials used in marine environments are subject to chemical, physical, and biological deterioration that can accelerate the degradation of structural materials. The corrosion behavior is one of the most destructive phenomena for marine structures steel and their alloys, and it is the electrochemical oxidation of the metal [1]. In the presence of chloride anion, the pitting corrosion causes failure in equipment due to the damage, but it only produces a small percentage of weight loss of the structure [2]. It is very common that the pits are difficult to detect because they have a very small size and they are covered with corrosion products [3,4]. The hole begins its formation when there is a defect in a protective film (oxide or organic coating), that results in the formation of an electrochemical cell. Moreover, this is an autocatalytic process that occurs within the hole. Therefore, the corrosion process produces suitable conditions for continuous activation of the pits and an increase of their depth [4,5,6]. On the other hand, some strategies have been developed to control the corrosion phenomenon, i.e., cathodic protection, employing either a sacrificial anode or an external power supply, anodic protection, employing a protective passive (oxide) layer on the metal surface, anodic and/or cathodic inhibitors, usually small organic molecules, used on the metal surface to impede either oxidation of the metal (anodic inhibitor) or the reduction reaction (cathodic inhibitor) [7]. The most viable option is preventing interaction (physical barrier) between the metal substrate (anode) and the dissolution that contains the corrosion agents (electrolyte) [8].

The efforts to mitigate the corrosion on steel and their alloys have been focused on the development of different types of coatings that intended to form a continuous film over the metallic surface, their function is to isolate the metal from direct contact of the surrounding electrolyte and to interpose high electrical resistance which prevents electrochemical reactions from occurring readily. All coatings, regardless of overall quality, could contain pores and holes that, in some cases, can be produced during application, transport or the installation of the metal parts [9].

Organic coatings have been extensively applied for protecting metallic substrates to retard the corrosion degradation in different media [10]. Among the organic coatings, the polyacrylates are interesting due to their properties, in particular the poly(methyl methacrylate) (PMMA) [5,6,11] Among various polyesters substrates, PMMA is still the main thermoplastic material used for diverse applications, such as optoelectronics, aeronautic, dentistry, microfluidic, household, military and many more engineering and industrial applications/purposes, since it possesses excellent weatherability, good transparency, high impact resistance (hardness), antireflective, low birefringence good processability (acrylic group can be hardened by heat and light when used as film) and also has compatible potential for being used with the organic matrix [12,13,14,15,16,17]. However, the application is limited due to its low corrosion resistance and thermal degradation. Regarding their properties, it is possible to enhance them with the addition of different materials, such as carbon or silicon dioxide [6,11,18,19,20,21].

In recent works, incorporation of small amounts of particles into polymers, such as hybrid organic/inorganic coatings, lead to improvements in thermal stability, mechanical, barrier but above all, anticorrosive properties [22], for example, the incorporation of carbon in the PMMA matrix as graphene [5] and carbon nanotubes [6] increases the thermal, mechanical and corrosion properties. Low density and high surface area were the most important properties of graphene that promote hydrophobicity of the hybrid coating that enhances anticorrosive properties and expands the potential application [5]. Carbon structures as carbon nanotubes (CNTs) also have been used to develop composite materials like hybrids coatings owing to their higher thermal and mechanical stability provides different properties. Moreover, the electrochemical measurements exhibited a high anti-corrosion resistance [6].

Silicon dioxide (SiO_2_) has been added into a PMMA matrix as an effort to improve the different properties of PMMA coatings such as mechanical resistance, thermal resistance, and adhesion as a result of the addition of silicon dioxide [6,18,21]. For example, multi-dome hydrophobic SiO_2_ nanoparticles (NPs), which have hydrophobic properties due to the arrangement of silica stacking layers [18]. Another way to incorporate SiO_2_ particles into the PMMA polymeric matrix is by their low corrosion using siloxane groups [6,20,21]. The increase in the adherence of PMMA coating is related to Si-O-metal bonds between siloxane and the metallic substrate [23]. This is an interesting system due to its transparency and chemical stability. Furthermore, the siloxane-PMMA coatings deposited on steel exhibit excellent corrosion resistance in saline media (3.5% NaCl) [6,21]. Therefore, to obtain hydrophobic coatings, PMMA-SiO_2_ that repels water creates huge opportunities in the area of corrosion protection for metals and alloys [19]. However, poor adhesion of the coating material to the steel, inhomogeneous particle distribution and superficial defects can cause not only delamination of the coating material, but the corrosion of the steel substrate [23,24].

In this work, hybrid coatings using a polymer matrix of PMMA and a different amount of commercial SiO_2_ nanoparticles were obtained to be deposited in a metallic sheet by using the dip-coating method. The barrier effect and hydrophobic properties of the coatings were corroborated using the electrochemical impedance spectroscopy (EIS) and polarization plots. Additionally, the hybrid coatings were tested with an artificial defect in their surface to corroborate the corrosion resistance and hydrophobic properties despite exposing the metallic substrate to the electrolyte. A better understanding of the artificial defect effect in the metallic surface is crucial for further improvement of the barrier properties of the material. In addition, the contact angle test was performed to corroborate the hydrophobicity of the pure PMMA and hybrid PMMA + SiO_2_ coatings.

## 2. Materials and Methods

### 2.1. Synthesis of the Coatings

#### 2.1.1. Synthesis of the (PMMA) Coating

The synthesis of the poly (methyl methacrylate) with a molecular weight of 300,000 was synthesized by free radical polymerization using freshly purified methyl methacrylate (Aldrich, contains ≤30 ppm MEHQ as inhibitor, 99%) and toluene (J. T. Baker, 99.98%) as solvent in 80:20 v/v ratio. The initiator was 2,2-Azo-Bis-Iso-Butyro-Nitrile (AIBN, Perkadox©, 99%) in a 1:1000 mol ratio against PMMA for the desired molecular weight. The polymerization was carried out at 95 °C with constant agitation for 90 min.

#### 2.1.2. Synthesis of the Hybrid PMMA Coating

Once prepared the PMMA was prepared, commercial SiO_2_ nanoparticles (AEROSIL R792 hydrophobic fumed silica after being treated with dimethylchlorosilane from Evonik), were added to obtain the hybrid PMMA. Previously, the SiO_2_ nanoparticles were dispersed in toluene for 5 min to obtain a homogenous solution and then added to the PMMA solution. The solution of the PMMA and SiO_2_ was mixed to disperse all the nanoparticles in the polymer and their incorporation was made using a cutting edge/high shear propeller at a speed of 14,000 rpm until the mixture was homogenous and no lumps were noticeable. The concentrations of the SiO_2_ nanoparticles were 0.01, 0.1% and 1% with respect to the concentration of the PMMA in the solution.

### 2.2. Stainless Steel Sheet Preparation and Deposition of the Coatings

The surfaces of the stainless steel AISI 304 sheets were prepared with a sandpaper mesh 240. After surface preparation, the deposition of PMMA and hybrid PMMA polymers was carried out using the dipping technique with homemade equipment that has a velocity rate from 0.1 to 99 mm per min. The immersion time of the metallic sheets was 5 min and an equal immersion/extraction rate that was 15 cm per min to obtain a polymer thickness of 90 ± 2 µm measured with a micrometer.

### 2.3. Physicochemical Characterization

The nanoparticle size, the distribution of SiO_2_ in the PMMA polymer matrix and the cross-section of the PMMA + SiO_2_ deposited on the stainless steel AISI 304 were carried out using a Hitachi SU8230 cold field emission scanning electron microscope (CFE-SEM, Hitachi, Tokyo, Japan) working at a low voltage of acceleration (3 keV).

In order to measure the contact angle, deionized water was used. Then 50 µL drop of the water was added to a sample and a digital image was taken using a Sony digital camera α99II coupled to a SLM50M28 Macro FE 50 mm lens (Sony Corporation, Tokio, Japan) under the same light conditions. Finally, the images were cropped to 680 × 680 pixels and then processed with a custom MATLAB (Mathworks Inc., Natick, MA, USA) script to obtain the data from each sample. The contact angle was measured five times in the same sample to obtain the standard deviation.

### 2.4. Electrochemical Characterization

#### 2.4.1. Electrochemical Impedance Spectroscopy

The electrochemical impedance spectroscopy (EIS) was collected in synthetic seawater (3.5 wt% NaCl solution) and all the electrochemical experiments were performed in a three-electrode electrochemical cell with both BioLogic VSP potentiostat and Gill AC potentiostat. Stainless steel AISI 304 sheets impregnated with the PMMA and hybrid PMMA polymers were used as working electrode, an Hg/Hg_2_Cl_2_ saturated with KCl was used as a reference electrode and a graphite rod was used as a counter electrode. The EIS response was obtained in a frequency range from 10^5^ to 10^−1^ Hz with an amplitude signal of 32 mV RMS and 10 steps per logarithmic decade for data acquisition. Moreover, the porous area and the capacitance of each PMMA coating were obtained from the experimental values using the Gill AC Sequencer (ACM Instruments Ltd., Cumbria, UK) and the Biologic EC Lab software v.10.31 (Biologic, Seyssinet-Pariset, France). The area of the working electrode that was exposed to the electrolyte was around 1 and 0.5026 cm^2^ for the coatings without artificial defect and with an artificial defect, respectively. All the metallic sheets coated with the PMMA and hybrid PMMA + SiO_2_ were tested with an artificial defect manually generated with the use of tip punch, with a diameter of approximately 0.5 mm, and without the artificial defect because it is necessary to study the behavior of the coating when it presents a failure.

#### 2.4.2. Polarization Curves

The PMMA and hybrid PMMA coatings were tested in synthetic seawater (3.5 wt% NaCl solution) to obtain the polarization curves (Tafel plots) and were performed in a three-electrode electrochemical cell with a Gill AC potentiostat. Stainless steel AISI 304 sheets impregnated with the PMMA and hybrid PMMA coatings were used as a working electrode, an Hg/Hg_2_Cl_2_ saturated with KCl were used as a reference electrode and a graphite rod was used as a counter electrode. The overpotential rage was from −1 to 1 V vs E_corr_. The area of the working electrode that was exposed to the electrolyte was 0.5026 cm^2^ for the coatings with an artificial defect manually generated with the use of tip punch, with a diameter of approximately 0.5 mm.

#### 2.4.3. Coating Performance by Scanning Electrochemical Microscopy Evaluation

The evaluation of the performance of the PMMA and PMMA + SiO_2_ coatings created, the scanning electrochemical microscopy (SECM, Biologic USECM470, Seyssinet-Pariset, France) technique was used. SECM is a powerful tool for obtaining localized information regarding corrosion on metal surfaces because it permits in situ characterization of topography and localized corrosion activity at microscale [25]. Measurements are performed recording electrochemical signals related to the interaction with the surface of a redox species in the solution phase [26], using a Biologic scanning system instrument model ac-SECM/SECM470, employing a four-electrode cell consisting of a platinum ultramicroelectrode (UME) of 25 µm diameter, a reference electrode of Ag/AgCl 3M KCl saturated, a graphite rod as an auxiliary electrode, and an ultra-microelectrode tip-to-sample distance of 19 µm was adjusted, which was obtained via the approach of curve recording in a 1 mM ferrocenemethanol (FcMeOH). A feedback model was used for SECM experiment, using a tip potential of 0.5 eV in order to detect the iron (II) species and obtain complete diffusion-limited electrochemical oxidation of FcMeOH to FcMeOH^+^ [25,26]. In addition, 1 mM FcMeOH was used as a mediator in a simulated seawater solution (composition) as electrolyte. The immersion time of the samples with the solution was 1 h.

## 3. Results and Discussion

### 3.1. Microstructural Characterization of SiO_2_ Nanoparticles and PMMA + SiO_2_ Coating

**Figure 1:**
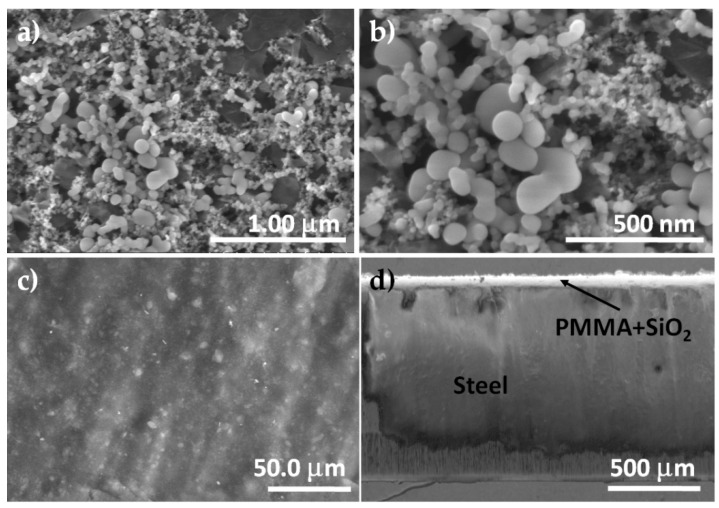
Scanning electron microscopy (SEM) micrographs of the morphology of: (**a**) and (**b**) commercial SiO_2_ particles, (**c**) poly (methyl methacrylate) (PMMA)-SiO_2_ coating, and (**d**) cross-section of the PMMA + SiO_2_ deposited on the stainless steel AISI 304.

The size and distribution of the commercial SiO_2_ nanoparticles were analyzed using scanning electron microscopy (SEM) and are shown in Figure 1a. From the SEM image, the morphology of the commercial SiO_2_ nanoparticles is not well defined, some particles seem like semi-spherical morphology but in most of the cases, the particles seem agglomerated. Additionally, the distribution of the particle size was from 20 to 150 nm (Figure 1b) but it is difficult to analyze because of the particles’ present agglomeration. The image of the surface of the PMMA matrix with the commercial SiO_2_ nanoparticles on the stainless steel AISI 304 sheets is presented in Figure 1c. The image shows many particles on the surface and embedded in the PMMA matrix, which indicates a good distribution of the particles in the PMMA matrix and also, an almost perfect coverage by the surface morphology is observed. The cross-section SEM image of the PMMA + SiO_2_ deposited on the stainless steel AISI 304 sheets (Figure 1d) shows a homogeneous layer with a thickness of approximately 82 ± 5 μm, which corresponds to measures obtained using the micrometer. It is important to mention that the coating does not present cracks or detachment, which indicates that it has very good adhesion.

### 3.2. Electrochemical Characterization of Hybrid PMMA + SiO_2_ Coating

**Figure 2:**
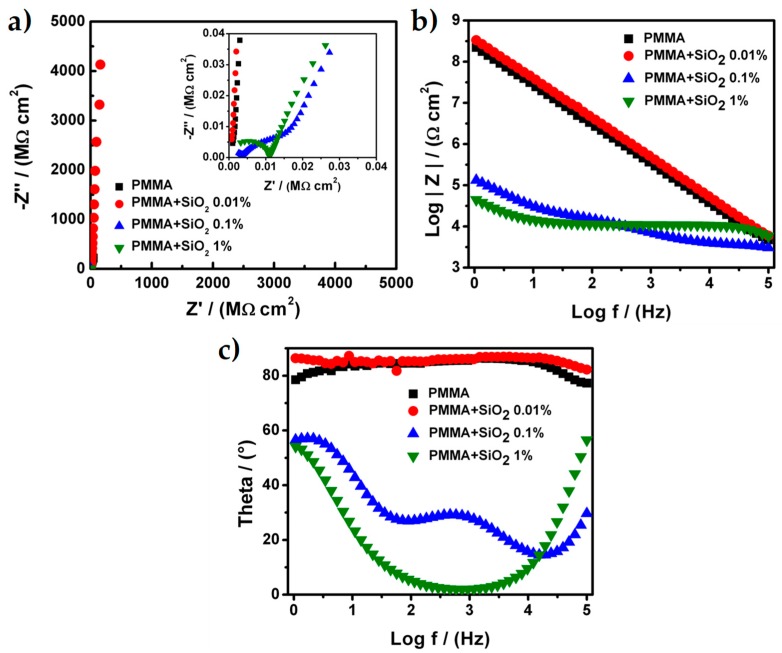
(**a**) Nyquist diagrams, (**b**) Bode diagrams and (**c**) phase-angle diagrams for the PMMA and PMMA + SiO_2_ coatings.

Once that the stainless steel AISI 304 sheets were prepared (polished) with a sandpaper 240 mesh, the PMMA hybrid coatings were prepared and evaluated in other to determine the effect of the addition of SiO_2_ nanoparticles with different concentration. Figure 2 shows the Nyquist and Bode diagrams for all samples of PMMA and PMMA + SiO_2_ nanoparticles that were used as coatings. The PMMA and PMMA + SiO_2_ 0.01% present, in both cases, only one process related with the capacitive behavior of the coating because the Nyquist diagram showed a straight line parallel at the impedance imaginary (Z’’) axis (Figure 2a). The total resistance for the PMMA and PMMA + SiO_2_ 0.01% coatings was 760 MΩ⋅cm^2^ and 247,800 MΩ⋅cm^2^, respectively. These values were higher and suggest that both coatings present a good adherence over the metallic sheet and in the case of the PMMA + SiO_2_ 0.01% coating the barrier effect was enhanced because the value of the total resistance was higher at less than two orders of magnitude when compared with the value of the total resistance of the PMMA coating.

For the Bode diagrams (Figure 2b), the modulus of the real impedance presents a constant increment along with the sweep frequencies and the results show a continuous line that corroborates only one process related to the capacitance of the coating. The maximum values of the modulus of the real impedance were 2.2 × 10^8^ and 3.3 × 10^8^ Ω⋅cm^2^ for PMMA and PMMA + SiO_2_ 0.01%, respectively, at the frequency value of 1 Hz. Additionally, the results of the theta angle (Figure 2c) exhibit a constant value between 75° to 85° for the PMMA and 85° for the PMMA + SiO_2_ 0.01%. These values corroborate the presence of one process associated with the capacitance of the coating. However, the Nyquist and Bode diagrams for the PMMA + SiO_2_ 0.1% and PMMA + SiO_2_ 1% coatings present at least two different processes: (1) The porosity of PMMA coatings, (2) the capacitance of PMMA coatings and (3) the resistance of the metallic sheet.

In the Nyquist diagram for the PMMA + SiO_2_ 0.1% coating the plot it seems that it presents only two processes, the porosity of the coating, the capacitance of PMMA coatings and the resistance of the metallic sheet. However, the Bode diagram exhibits three processes because the modulus of the real impedance has three different changes in its slope that are associated with the three different processes. The total resistance calculated of each process was 11.884 × 10^3^, 20.416 × 10^3^ and 2.402 × 10^3^ Ω⋅cm^2^ related with the porosity of PMMA coating, the capacitance of PMMA coating and resistance of the metallic sheet processes, respectively. Moreover, the Nyquist diagram for the PMMA + SiO_2_ 1% coating presents two different processes associated with the porosity of the PMMA coatings and resistance of the metallic sheet. The total resistance for each process was calculated and the values were 79.515 × 10^3^ and 10.727 × 10^3^ Ω⋅cm^2^. The Bode diagram validated the two different processes for the PMMA + SiO_2_ 1% coating due to the slope of the modulus of the real impedance. There are changes in the slope and the theta angle that clearly show two different values.

The results of total impedance, modulus of the real impedance, as well as the shape of the Nyquist and Bode diagrams, show that the addition of nanoparticles of SiO_2_ into the PMMA matrix improve the barrier effect over the coating but if the amount of SiO_2_ increases above 0.01% with respect to PMMA, the properties of the coating could be affected, such as the adherence of the coating on the metallic sheet that results from an increment of the porosity over the coating that, in some cases, exposes the metallic substrate to the electrolyte.

In order to corroborate the properties of all coatings, the value of the capacitance and the porous area were calculated. These results help to determine the enhancement of the barrier effect resulting from the addition of the nanoparticles of SiO_2_. The porous area for each coating was determined using the following Equation (1):

(1) $$A_{po}=\rho \frac{l}{R_{po}}$$

where $A_{po}$ is the porous area, ρ is the density of the electrolyte, l is the distance between the working electrode and the reference electrode and $R_{po}$ is the total resistance of the system obtained from the experimental values.

The capacitance of the PMMA and PMMA + SiO_2_ coatings was calculated using the software Biologic EC Lab v.10.31 of the potentiostat (Biologic, Seyssinet-Pariset, France). For the PMMA and PMMA + SiO_2_ 0.01% an electric circuit equivalent (ECE) array was used for a capacitive behavior which has a resistance (associated with the resistance of the electrolyte) and a capacitor connected in serie. However, for the PMMA + SiO_2_ 0.1% and PMMA + SiO_2_ 1% coatings, the data treatment was different because in these coatings, more than one process was present, changing the ECE array. All of the parameters used to calculate the porous area and the capacitance of each coating are shown in Table 1 were the R_E_ is the resistance of the electrolyte, the R_PRO_ is the total resistance in each process and the A_PO_ is the porous area.

**Figure 3:**
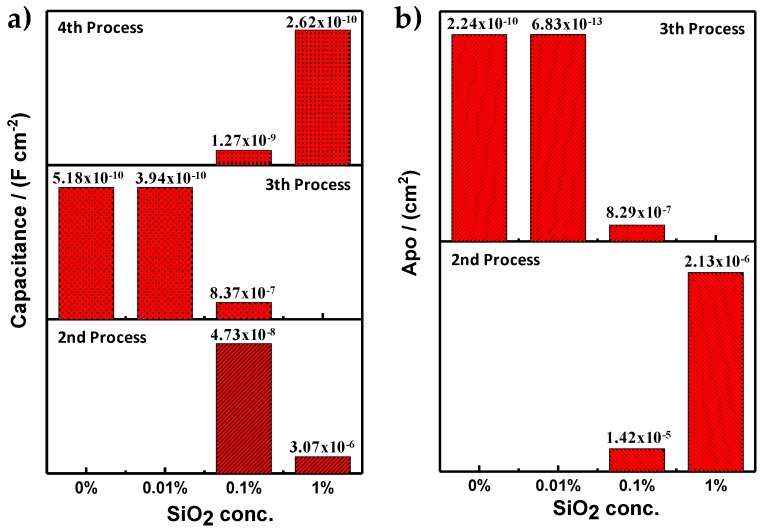
(**a**) Capacitance values and (**b**) porous area for the PMMA and PMMA with different concentrations of SiO_2_ nanoparticles.

In Figure 3a, the capacitance for the PMMA and PMMA + SiO_2_ coatings for the different processes above mentioned can be observed. The second process is related to the pores over the coating, the oxides in those pores and the possible presence of the cathodic delamination phenomena that could occur in cathodic or equilibrium potential [27,28,29]. The third process is related with the resistance/capacitance of the coating which provides information of coating integrity and the fourth process is related to the resistance/capacitance of the metallic substrate that is exposed to the electrolyte. It is important to remark that the PMMA and PMMA + SiO_2_ 0.01% coatings exhibit the lowest values of capacitance. In this particular case, the PMMA + SiO_2_ 0.01% coating presents the lowest value of capacitance (3.94 × 10^−10^ F⋅cm^−2^) that is directly associated with high resistance of the coating. Moreover, as was mentioned before, the PMMA + SiO_2_ 0.01% coating shows only one process (third process, the resistance/capacitance of the coating). For the results of the porous area (Figure 3b), PMMA + SiO_2_ 0.01% coating obtained the lowest value (6.8 × 10^−13^ F⋅cm^-2^). These results suggest that the addition of the nanoparticles of SiO_2_ at 0.01% to the PMMA matrix enhance the barrier effect because the capacitance and the porous area for the third process were the best values that were obtained.

**Figure 4:**
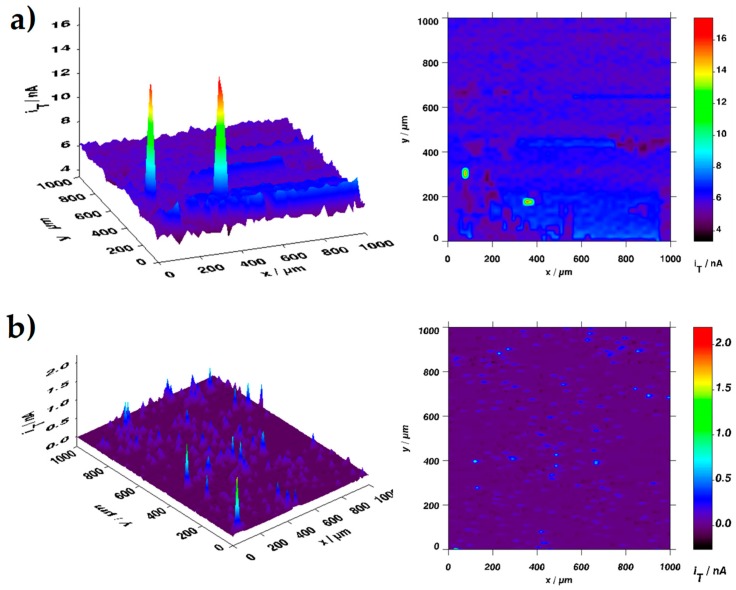
SECM images of: (**a**) PMMA and (**b**) PMMA + SiO_2_ 0.01% coatings. The current of the peaks is related with defects on the surface of the coatings.

Scanning electrochemical microscopy (SECM) analysis was carried out to determine the integrity of the coating. If the coating presents any defect on its surface, there are variations of the current peak that could be associated with different thickness on the coating, a higher pore density (higher values of porous area) or in the worst case, a corrosion process because the metallic substrate is exposed to the electrolyte [30]. Furthermore, the results that were obtained for the SECM have a direct relation with the adherence and the integrity of the coatings. For the SECM analysis, only PMMA best-obtained coatings were used. Figure 4 shows the SECM images for the PMMA and PMMA + SiO_2_ 0.01% coatings. In both images, the SECM analysis exhibits current peaks in the surfaces of the coatings with a setting potential of 0.50 V vs. Ag/AgCl 3M KCl saturated to determine the behavior of the iron II species in the electrolyte and their interaction with the metallic surface and the coating [26]. The approximations of the microdisk tip to the surface of the samples for this work were carried out with an experimental calibration curve using the molecule probe (ferrocenemethanol), however, there is a very good theoretical approximation that enhances the current values for the approximation of the microdisk tip [31,32].

The results for the samples show current peaks were related to a difference in the coating thickness or discontinuities over the coatings that could expose the metallic substrate to the electrolyte. However, the values of the current peaks were in the magnitude order of nano-Ampere (nA) discarding a process related to a corrosion phenomenon because the values of the resistance of the PMMA coatings were higher resulting in lowest values of the current along with the measurement. For the PMMA coating (Figure 4a) it could be observed that the surface exhibited different values of the current from 5 to 7 nA that show a good continuity of the PMMA coating over the metallic sheet. However, the images present two peaks with a higher value of current (16 nA) which could be related with the percolation of the electrolyte into the PMMA coating owing the pores that were present in the polymer thickness or a punctual bad adherence of the polymer coating to the metallic substrate increasing the concentration of the iron II resulting in an increment of the current values over the PMMA coating surface. Regarding the PMMA + SiO_2_ 0.01% coating (Figure 4b), the higher values of the current throughout the analysis area were around 1 nA but the images show that in several zones of the analysis area the current exhibited values are around 0 nA. These results are related with the decrease in the amount of the electrolyte that could be percolated into the coating thickness because the barrier effect increased due to the addition of the SiO_2_ particles to the polymer matrix and resulting in an improvement of the hydrophobicity of the PMMA + SiO_2_ 0.01% coating without affecting the adhesion of the polymer over the metallic sheet

The results that were obtained with the SECM technique for the PMMA + SiO_2_ 0.01% coating present a direct relationship with the higher values of the resistance obtained in the impedance measurement and with the lower values of the porous area were calculated resulting in an enhancement of the properties of the coating with the addition of the SiO_2_ particles into the polymeric matrix.

### 3.3. Electrochemical Characterization of PMMA and Hybrid PMMA + SiO_2_ Coating with an Artificial Defect

**Figure 5:**
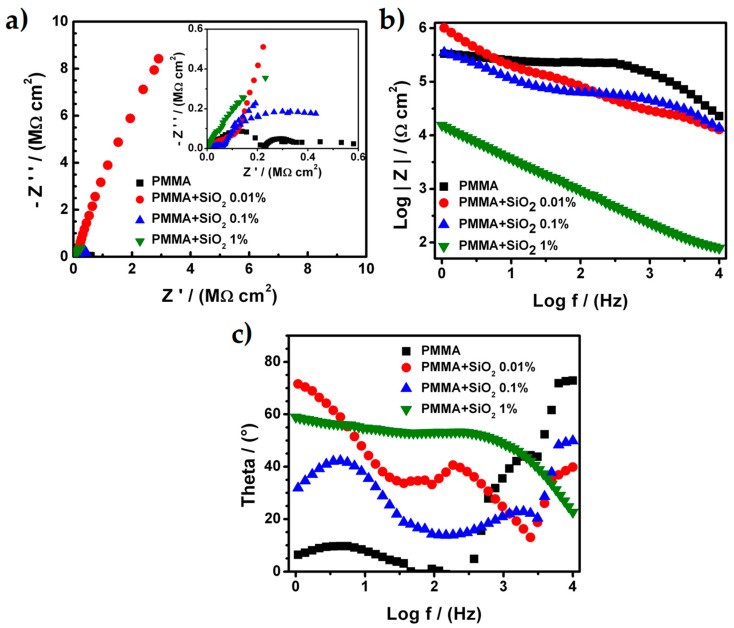
(**a**) Nyquist diagrams, (**b**) Bode diagrams and (**c**) phase-angle diagrams for the PMMA and PMMA+SiO_2_ coatings with an artificial defect on the surface.

During application, transport or installation of the metal parts, the coatings can be damaged. Therefore, sample performance was evaluated with an artificial defect, exposing the metallic substrate to the electrolyte. The SiO_2_ nanoparticles, in that case, could increase the barrier effect in the coating due to their hydrophobic properties. Figure 5 shows the Nyquist and Bode diagram for PMMA and PMMA + SiO_2_ coatings with an artificial defect. The processes found in this case were four. The first process was related with the resistance/capacitance of the artificial defect, the second process was related with the pores over the coating, the oxides in those pores and the possible presence of the cathodic delamination phenomena [33,34]. The third process was related to the resistance/capacitance of the coating and finally, the fourth process was related to the resistance/capacitance of the metallic substrate exposed to the electrolyte. The PMMA coating with the artificial defect on the surface presented several changes in the Nyquist diagram (Figure 5a) in comparison with the Nyquist diagram without an artificial defect (Figure 2a). In this case, the Nyquist diagram shows two different processes: The related with the resistance/capacitance of the artificial defect (first process) and the related to the resistance/capacitance of the metallic substrate that is exposing to the electrolyte (fourth process). At high frequencies, could be two processes, but if the total resistance values are of the same magnitude order (1.01 × 10^5^ and 1.76 × 10^5^ Ω⋅cm^2^), then it is only one process. The total resistance for the fourth process is 1.37 × 10^5^ Ω⋅cm^2^. For the PMMA + SiO_2_ 0.01% coating with the artificial defect, the Nyquist diagram shows a capacitance process similar to the previous results except for the value of the impedance, which is lower, and this behavior is related with the process associated with the resistance of the coating. Moreover, the values remain in the magnitude order of megaohms. However, if the impedance scale changes in the plot to lower values, another two processes can be observed. The first process is related to the resistance/capacitance of the artificial defect and the second process are related with the pores over the coating, the oxides in those pores and the possible presence of the cathodic delamination phenomena. In both cases, the two processes were incomplete due to the resistance of the PMMA + SiO_2_ 0.01% coating and the process that predominated was the resistance/capacitance of the coating.

Regarding the Bode diagram (Figure 5b) and theta angle (Figure 5c), the results exhibit three different processes: The process related with the resistance/capacitance of the artificial defect (first process), the process related with the pores over the coating, the oxides in those pores and the possibile presence of the cathodic delamination phenomena (second process) and the process related with the resistance/capacitance of the coating (third process). The value of the total resistance for each process was 2.11 × 10^4^, 1.46 × 10^5^, and 2.97 × 10^7^ Ω⋅cm^2^ from first, second, and third process, respectively. It is important to notice that the resistance for the coating (third process) is high compared with its capacitive behavior shown in the Nyquist diagram, and it is the unique coating that conserved the resistance in magnitude order of megaohms. Moreover, the PMMA + SiO_2_ 0.1% coating presents three processes that are clearly identified in the Nyquist and Bode diagram. Those three processes are: The process related with the resistance/capacitance of the artificial defect (first process), the process related with the pores over the coating, the oxides in those pores and the possibility presence of the cathodic delamination phenomena (second process) and the related to the resistance/capacitance of the metallic substrate that is exposed to the electrolyte (fourth process).

It is evident that the amount of 0.1% of SiO_2_ nanoparticles with respect to PMMA does not allow a good adherence of the coating or cathodic delamination that promotes the percolation of the electrolyte to the metallic substrate. Finally, the Nyquist and Bode diagrams for the PMMA + SiO_2_ 1% coating presents only one process associated with the resistance/capacitance of the metallic substrate that is exposing to the electrolyte (fourth process). For this case, the amount of 1% of SiO_2_ nanoparticles promotes the percolation of the electrolyte to the metallic substrate.

**Figure 6:**
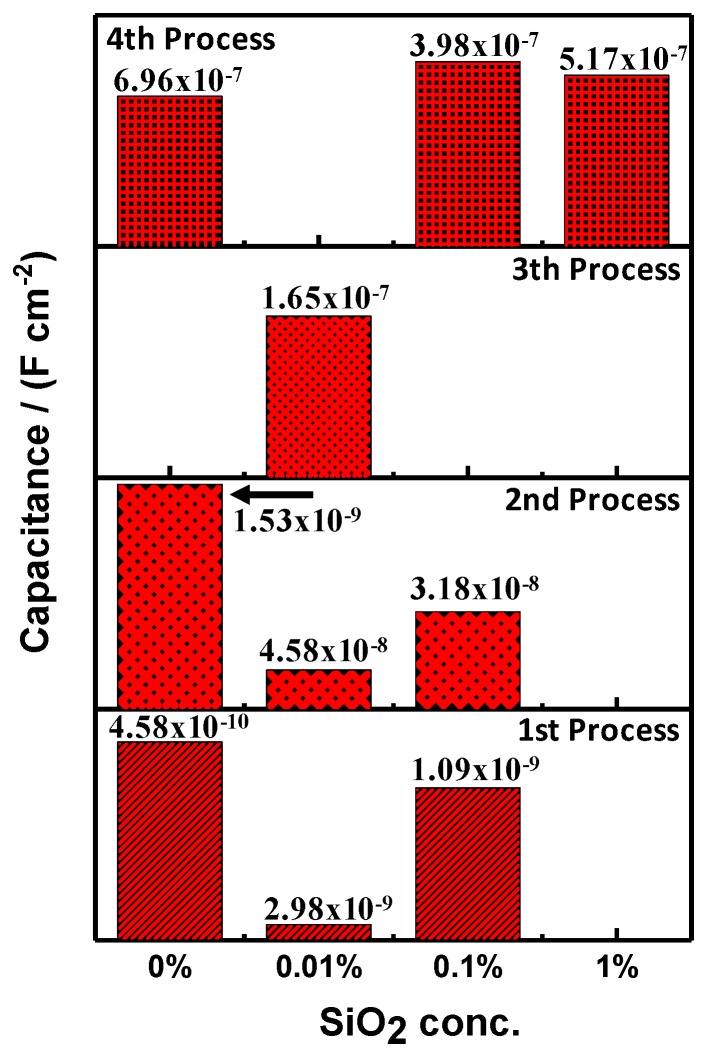
Capacitance values for the PMMA and PMMA with different concentrations of SiO_2_ nanoparticles with an artificial defect on the surface.

The four different processes that appeared on the coating samples with an artificial defect can be corroborated with the capacitance of each process (Figure 6). It is important to notice that the PMMA + SiO_2_ 0.01% coating is the unique sample in which the fourth process did not exist, this means that the electrolyte did not get in contact with the metallic substrate, and the first and second processes were identified in the measurement. Coating performance is promising because it continues functioning even with the artificial defect. Furthermore, the PMMA coating shows the resistance/capacitance of the pores and also the resistance/capacitance of the metallic surface, because it does not present the hydrophobicity behavior as, in this case, SiO_2_ nanoparticles were not added. Finally, the amount of SiO_2_ nanoparticles decrease the adherence of the PMMA + SiO_2_ 0.1% and PMMA + SiO_2_ 1% coatings over the metallic surface and promotes an increase of pores where the electrolyte can percolate and, in the worst case, can totally expose the metallic surface. Furthermore, the calculated values related with the resistance of the electrolyte (Table 2) show the higher resistance was for the PMMA + SiO_2_ 0.01% coating suggesting that the barrier effect in addition to the hydrophobic properties of the SiO_2_ particles in the PMMA matrix decreased the percolation of the electrolyte which avoided the corrosion of the metallic substrate (fourth process) despite the artificial defect over the surface coating.

**Figure 7:**
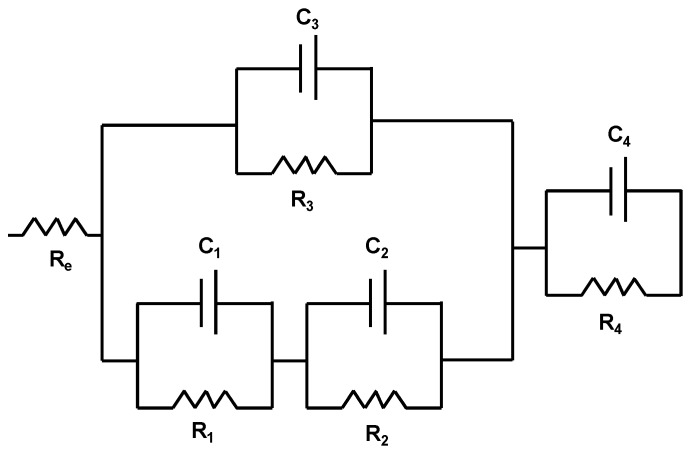
Electrical equivalent circuit model used to simulate EIS data for the PMMA + SiO_2_ coatings.

An electrical equivalent circuit model (Figure 7) based on the four different processes that were determined in the different coatings is proposed. The Re correspond to the resistance of the electrolyte, if the coating presents an artificial defect in the first process their resistance/capacitance effect (C_1_ and R_1_) can be observed. The second process related to the pores over the coating, the oxides in those pores and the possible presence of the cathodic delamination phenomena can be represented with the resistance/capacitance array C_2_ and R_2_. In parallel array to the first and second process appear sthe third process related to the coating and represented by C_3_ and R_3_. Finally, the fourth process is related to the metallic substrate in a series array which respects the other processes and is represented by C_4_ and R_4_.

**Figure 8:**
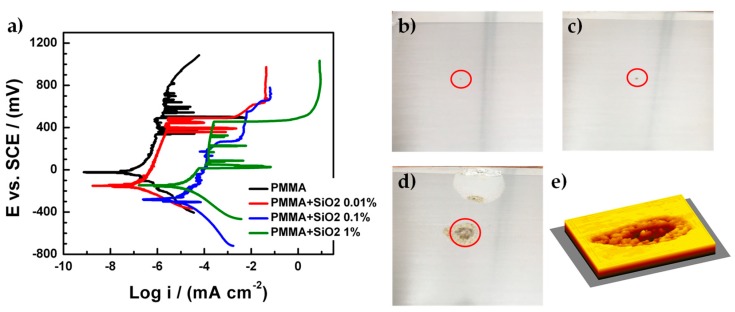
(**a**) Polarization curves of the PMMA coatings and images of the metallic sheets cover for: (**b**) PMMA + SiO_2_ 0.01%, (**c**) PMMA + SiO_2_ 0.1%, (**d**) PMMA + SiO_2_ 1% coatings, and (**e**) 3D image of the affected zone for the corrosion process.

The corrosion rate and the corrosion potential were determined from the polarization curves (Figure 8a) for all the coatings with the presence of an artificial defect on their surface. For all the coatings the pitting corrosion process on the metallic substrate can be observed because the current densities exhibited abrupt changes in their values from log −6 to log −2, approximately, for the cases of the PMMA and PMMA + SiO_2_ 0.01% coatings but the pitting corrosion process was presented at high potentials (400 mV vs. SCE) compared to PMMA + SiO_2_ 0.1% and PMMA + SiO_2_ 1% coatings. This behavior is related to the hydrophobicity of the PMMA and PMMA + SiO_2_ 0.01% coatings due to the SiO_2_ particles that promote the protection of the metallic substrate from the electrolyte.

The corrosion parameters of the PMMA coatings are shown in Table 3. The corrosion potential values (E_corr_) corresponding to the PMMA, PMMA + SiO_2_ 0.01%, PMMA + SiO_2_ 0.1% and PMMA + SiO_2_ 1% coatings were −24, −155, −228, −144 vs. SCE, respectively. The best result for the corrosion rate was obtained for the PMMA + SiO_2_ 0.01% coating since it is the lowest value in corrosion rate, compared with the other coatings, although it seems that the PMMA + SiO_2_ 0.1% coating presents a corrosion potential of −228 mV vs. SCE, it is difficult to determine due to the cathodic delamination phenomenon that was clearly observed, in this case, the current density presents a shifting to lowest values near the corrosion potential where it is supposed to appear [27,28]. These results of the corrosion rate and the corrosion potential corroborate the increase in the hydrophobic capacity of the hybrid coating PMMA + SiO_2_ 0.01% despite the presence of an artificial defect on the coatings that expose the metallic substrate to the electrolyte.

Figure 8b–d shows the images of the metallic sheets covered by hybrid PMMA coatings with an artificial defect on their surface after electrochemical impedance spectroscopy and polarization curves tests. The artificial defects on coatings were located inside red circles. It can be observed that in the PMMA + SiO_2_ 0.01% coating (Figure 8b), the metallic sheet does not have corrosion products inside the artificial defect. However, the PMMA + SiO_2_ 0.1% coating (Figure 8c) has corrosion products into the artificial defect as well as the PMMA + SiO_2_ 1% coating (Figure 8d) with the most affected zone for the corrosion process. Figure 8e shows a 3D image of the affected zone from the PMMA + SiO_2_ 1% coating sample. From the image the cavity or hole produced in the material can be observed, which produces mechanical damage to the protective coating. The results are directly related with the amount of SiO_2_. If the amount of SiO_2_ increases, the adherence of the PMMA matrix decreases, resulting in an increase of the electrolyte that percolated derived in a major damage zone.

**Figure 9:**
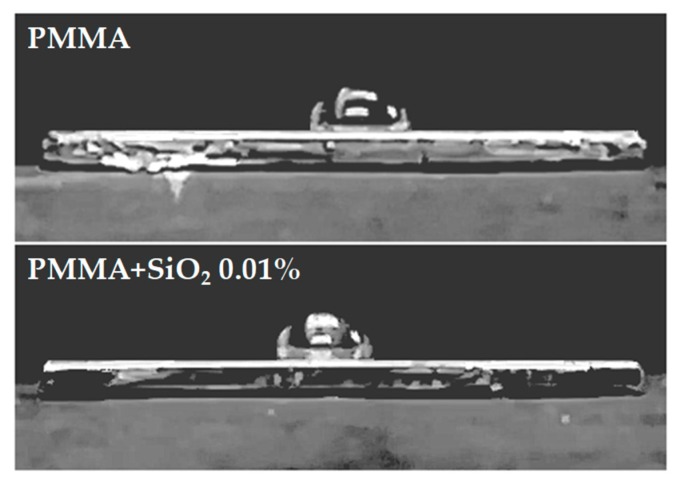
Water contact angle measurements performed on PMMA and PMMA + SiO_2_ 0.01%.

Finally, to corroborate the hydrophobicity of the best coatings (PMMA and PMMA + SiO_2_ 0.01%) the deionized water contact angle was determined (Figure 9). The water droplet on coatings PMMA surfaces is round and has larger contact angles. As is shown in Table 4, the value of contact angle for PMMA + SiO_2_ 0.01% coating is high compared with the PMMA, confirming that the addition of SiO_2_ nanoparticles to the PMMA matrix provides an increase in the hydrophobicity of the coating. Therefore, the surface energy of the PMMA + SiO_2_ 0.01% surface is low, it has a poor wetting and poor adhesiveness of the drop, resulting in a larger contact angle (around 90°). These results agree with the previously reported [35], where an increase in the resultant water contact angle values (a better hydrophobic behavior) makes possible an increase in the corrosion resistance of the coating.

## 4. Conclusions

According to the results, the addition of the SiO_2_ nanoparticles to the polymeric matrix of PMMA increases the barrier effect and the hydrophobicity of the coatings using an amount of 0.01% keeping up a good adherence. This result was corroborating with the Nyquist diagram that showed a capacitive behavior with a total resistance of 247,800 MΩ⋅cm^2^. Moreover, in the Bode diagrams, the maximum value for the modulus of the real impedance was 3.3 × 10^8^ Ω⋅cm^2^ at the frequency value of 1 Hz and the results of the theta angle exhibit a constant value of 85°. Furthermore, the capacitance calculated was 3.9 × 10^−10^ F⋅cm^-2^ being the lowest value and the porous area was lower (6.8 × 10^−13^ cm^2^). Those results corroborate the presence of one process (third process) associated with the capacitance of the coating and excellent adherence to the metallic substrate. When the PMMA coatings present an artificial defect on their surface, the PMMA + SiO_2_ 0.01% coating exhibited the best results again due to the Nyquist diagram shows a capacitance process similar to the Nyquist diagram without an artificial defect but, in this case, the value of the impedance decreases. However, these values are still in the magnitude order of megaohms. Regarding the Bode diagram, the results exhibit first, second and third process with a total resistance value of 2.11 × 10^4^, 1.46 × 10^5^, and 2.97 × 10^7^ Ω⋅cm^2^, respectively. Those are excellent results because the performance of the coating with the artificial defect were similar to the coating without an artificial defect besides that we exposed the metallic surface to the electrolyte. These results suggest that the addition of SiO_2_ nanoparticles (0.01%) enhance the PMMA properties as the barrier effect and hydrophobic behavior despite the presence of a defect that exposes the metallic substrate to the electrolyte. Based on the results, hybrid PMMA + SiO_2_ coatings can be considered as very promising candidates to protect metallic surfaces from corrosion phenomenon of devices for marine environments.

## Figures and Tables

**Table 1:** Electrochemical impedance spectroscopy (EIS) parameters for the PMMA and PMMA + SiO_2_ coatings in synthetic seawater.

Sample	Process	R_E_ (Ω⋅cm^2^)	R_PRO_ (Ω⋅cm^2^)	Capacitance (F⋅cm^−2^)	A_PO_ (cm^2^)
PMMA	3th	1372	7.55 × 10^8^	5.18 × 10^−10^	2.24 × 10^−10^
PMMA + SiO_2_ 0.01%	3th	1060	2.48 × 10^11^	3.94 × 10^−10^	6.83 × 10^−13^
PMMA + SiO_2_ 0.1%	2nd	1920	11.88 × 10^3^	4.73 × 10^−8^	1.42 × 10^−5^
	3th	-	204.167 × 10^3^	8.37 × 10^−7^	8.29 × 10^−7^
	4th	-	2.402 × 10^3^	1.27 × 10^−9^	-
PMMA + SiO_2_ 1%	2nd	584	79.514 × 10^3^	3.07 × 10^−6^	2.13 × 10^−6^
	4th	-	10.727 × 10^3^	2.62 × 10^−10^	-

**Table 2:** EIS parameters for the PMMA and PMMA + SiO_2_ coatings in synthetic seawater with an artificial defect on the surface.

Sample	Process	R_E_ (Ω⋅cm^2^)	R_PRO_ (Ω⋅cm^2^)	Capacitance (F⋅cm^−2^)
PMMA	1st	1803	9.55 × 10^4^	4.58 × 10^−10^
	2nd	-	1.91 × 10^5^	1.53 × 10^−9^
	4th	-	1.37 × 10^5^	6.96 × 10^−7^
PMMA + SiO_2_ 0.01%	1st	11.158 × 10^3^	2.11 × 10^4^	2.98 × 10^−9^
	2nd	-	1.46 × 10^5^	4.58 × 10^−8^
	3th	-	2.97 × 10^7^	1.65 × 10^−7^
PMMA + SiO_2_ 0.1%	1st	965	2.86 × 10^4^	1.09 × 10^−9^
	2nd	-	4.82 × 10^4^	3.18 × 10^−8^
	4th	-	4.82 × 10^5^	3.98 × 10^−7^
PMMA + SiO_2_ 1%	4th	63	72.8	5.17 × 10^−7^

**Table 3:** Corrosion parameters for the PMMA coatings in 3.5 wt% NaCl solution.

Sample	*E_corr_* (mV)	*i_corr_* (mA·cm^−2^)	Corrosion Rate (mmpy)
PMMA	−24	3.258 × 10^−7^	3.783 × 10^−6^
PMMA + SiO_2_ 0.01%	−155	3.090 × 10^−7^	3.588 × 10^−6^
PMMA + SiO_2_ 0.1%	−228	-	-
PMMA + SiO_2_ 1%	−144	2.472 × 10^−5^	2.870 × 10^−4^

**Table 4:** Values of the contact angle for the PMMA coatings.

Sample	Contact angle (°)
PMMA	84.71 ± 0.82
PMMA + SiO_2_ 0.01%	87.06 ± 0.78
